# Perceptions of barriers and levers of health-enhancing physical activity policies in mid-size French municipalities

**DOI:** 10.1186/s12961-020-00575-z

**Published:** 2020-06-08

**Authors:** A. Noël Racine, J. M. Garbarino, K. Corrion, F. D’Arripe-Longueville, B. Massiera, A. Vuillemin

**Affiliations:** Université Côte d’Azur, LAMHESS, Nice, France

**Keywords:** Health-enhancing physical activity, local policy, municipality, policy-making, concept mapping

## Abstract

**Background:**

Policy is one of the levers for initiating structural change to foster the promotion of health-enhancing physical activity (HEPA). To this end, policy-makers have to deal with complex ecosystems embedded in specific contexts. However, limited research has been conducted on this topic at the local level. The purpose of this study was to identify the perceived barriers and levers of HEPA policies according to department heads and elected officials across various sectors in mid-size French municipalities.

**Methods:**

This study used a mixed method primarily based on an adaptation of the concept mapping approach. A list of statements completing the sentence: ‘In a mid-size municipal context, to develop HEPA policies, it is necessary to …’ was submitted to key informants of 17 mid-sized French cities. Key informants in each municipality first rated the importance of each statement without considering their local context; they then rated the feasibility of each statement given their local context. In both cases, they used a Likert scale ranging from 1 to 6.

**Results:**

A total of 23 municipal department heads and 10 elected officials from the sport (*n* = 14), health (*n* = 10) and social (*n =* 9) sectors in 11 mid-size French cities participated in this study. A list of 84 statements, sorted into 16 categories, was rated by participants according to their importance (*M =* 4.52, *SD =* 0.86) and their feasibility (*M =* 3.77, *SD =* 0.74). Potential barriers to (*n =* 10) and levers of (*n =* 38) HEPA policy development were identified. These results varied according to the position and sector of the participants.

**Conclusions:**

The results suggest that local contextual factors can affect the development of HEPA policies in mid-size French municipalities. The different perceptions of the potential levers and barriers according to sector might affect intersectoral collaboration. This study contributes by enhancing understanding of how local HEPA policies are developed in the French context.

## Background

Physical inactivity and sedentary behaviours are important risk factors for chronic diseases [1]. These issues have thus become a preoccupation in the public health policy field [2]. Over the past several years, governments at international, national and local levels have been developing policies to promote health-enhancing physical activity (HEPA) [3–6]. According to the literature, policy is one of the levers for initiating structural change to address the issues related to physical inactivity and sedentary behaviours [7]. Policy can indeed influence many of the social, economic and physical health determinants [8, 9]. Influenced by these determinants, the environments in which people live have a particularly strong influence on their health behaviours [10], including physical activity [11, 12]. Yet, in order to have a significant influence on health behaviours, polices should be intersectoral, following the Health in All Policies approach [13, 14]. This approach consists of taking health decisions across a range of policy areas outside the health sector and the local level is particularly important for this policy implementation [15–18]. In France, the decentralisation process has included the transfer of some of the legal and ‘facultative’ competencies (optional according to the law) from the national government to local governments [19]. This has meant that local governments can use their competencies to act on many health determinants such as urban, environmental, social, sport and health factors [4]. From these competencies, municipalities have the authority to influence the conditions and the environment where people live; it is a particularly suitable level to promote an active and healthy lifestyle [20, 21]. Studies on national HEPA policies also highlighted the importance of the local level [22, 23]. To this end, municipalities play a major role in implementing intersectoral policies for HEPA promotion [4]. However, implementing intersectoral policies can be difficult [18] as policy-makers from various sectors need to share a common language, vision and policy goals [18]. Involvement in intersectoral policies may be limited when policy-makers lack an understanding of the benefits of this approach and have different policy priorities based on their sector [18, 24]. Their perceptions of the importance of HEPA policies, especially when they come from different sectors, may be an indicator of their willingness to become involved [25, 26]. Studies have shown that capturing the views of policy-makers and professionals across multiple sectors therefore provides a deeper understanding of the key factors that facilitate intersectoral collaborations and HEPA policy implementation [27, 28]. However, there is still a lack of local evidence on HEPA promotion to help governments in their policy decisions [29], whereas contextual variables might well have an important influence on local HEPA policy development [30]. Thus, this study sought to (1) capture the perceptions about HEPA policy development from municipal department heads and elected officials in different sectors and (2) identify the barriers and levers of HEPA policies according to the local context.

## Methods

### Participatory mixed method

This study was conducted following a participatory mixed method mainly based on an adaptation of the concept mapping approach (CMA) [31]. Based on qualitative data and statistical analysis, CMA can be used to explore, capture and compare the perceptions of different types of stakeholders [32, 33], including the perception of their barriers and facilitators about a specific topic [34, 35], which is in line with our concerns in this study. Moreover, CMA seems to be particularly promising to develop evidence-based strategies in the public health policy field [36]. The CMA is a 6-step process described by Trochim [37]. Which involves the preparation, the generation of statements, the structuring of statements, the representation of statements, the interpretation of maps and the utilisation of maps. However, this is not the only way to accomplish CMA. Some studies have adapted the technique of CMA “*to meet specialised needs and to accommodate external constraints*” [38]. In this study, the characteristics and the constraints of the targeted participants impacted the design of this study and prompted researchers to adapt the technique of CMA. Thus, the following adapted four steps were used: (1) preparation, (2) generation of statements, (3) participant selection and statement rating, and (4) mapping analysis. The Concept Systems Global Max^©^ software [39] was used for the mapping analysis step.

### Preparation

The preparation step involved defining the focus prompt sentence that could be formulated in an open-ended way. Researchers in the physical activity for health domain (*n =* 6) formulated the following: ‘In a mid-size municipal context, to develop health-enhancing physical activity policies, it is necessary to …’.

The key terms of the focus prompt sentence, like ‘policy’ and ‘physical activity’, were agreed upon and defined by the researchers. Policy was defined as “*legislative or regulatory action taken by federal, state, city, or local governments, government agencies, or nongovernmental organizations. Policy includes formal and informal rules and design standards that may be explicit or implicit*” [40]. Physical activity was defined as follows: “*any bodily movement produced by skeletal muscles that requires energy expenditure, it can include sport and any physical practice in daily living*...” [41].

### Generation of statements

In the CMA, the generation of statements step is usually completed by the same group of participants who then sort and rate these generated statements. In this study, researchers faced the challenge of involving policy-makers as participants (i.e. elected officials and department heads) with ‘specific constraints’. This type of participant had a limited time available to participate in a study with several rounds. Managing the recruitment process can be difficult in CMA with certain types of participants [42]. Moreover, the number of participants may decrease throughout the CMA steps due to lack of availability, attrition or fatigue to several rounds of participation [43]. Considering this, a group of experts (*n* = 12) was constituted for the statement-generation step and sorted these statements into themed categories, whereas a group of policy-makers (*n* = 33) was recruited for the rating step to collect more quantitative data for the analysis. Thus, this strategy reduced the risk of losing participants throughout the process and increased the chances of collecting more quantitative data for analysis.

The generation of statements was based on the focus prompt sentence defined in the preparation step. The aim was to integrate scientific and practical knowledge in the generated statements. The group of experts included researchers in the physical activity for health domain (*n =* 6), municipal department heads (*n =* 3) and elected officials (*n =* 3). Department heads and elected officials were selected by the research team from municipalities strongly engaged in HEPA promotion. First, the researchers conducted a literature review to identify the key determinants and key factors for developing HEPA policies in a municipal context. PubMed, Web of Science, ScienceDirect and Google Scholar databases were used to search for the terms ‘physical activity’, ‘policy’, ‘local government’ and ‘municipality’ in English and French between 2007 and 2018. Following extraction of the relevant scientific literature on the topic, a first list of statements was generated by the researchers to complete the focus prompt sentence.

Based on their experiences, municipal department heads and elected officials generated a second list of statements. The first and second lists of statements were then merged to obtain a single list. From there, other brainstorming sessions (*n* = 4) were organised with the group of experts. During these sessions, the statements were classed in order to build categories of determinants. The statements and categories were added, deleted or redefined until a final consensus was reached. Moreover, duplicate ideas were removed, and the wording of the statement elements was enhanced to improve clarity. After the brainstorming sessions, a final list of statements sorted into categories was generated. Thus, the group of experts deliberately decided not to invite policy-makers to sort the statements into piles using Concept Systems Global^©^ software. Instead, consensus was found through brainstorming sessions, the group of experts decided to sort statements into categories that would be understandable and relevant, and therefore easier to rate by the group of policy-makers. Sorting is usually used to measure, analyse and map the relationship as well as the perceived similarity between statements [31]. However, this was not the objective in this study.

### Participant selection and statement rating

#### Participant selection

Participants were selected from 17 mid-size municipalities (between 20,000 and 100,000 residents [44]) from the Alpes-Maritimes and Var counties in France. These two counties, in close proximity to the research team, were selected to facilitate data collection. Small municipalities (under 20,000 inhabitants according INSEE [44]) have less resources to develop HEPA policies compared to mid-size municipalities. Thus, policy-makers might not have the same experience and perception to HEPA policy development. In these counties, there are only two big municipalities (over 100,000 inhabitants according INSEE [44]) with a different magnitude of resources compared with mid-size municipalities; thus, to ensure more homogeneous municipalities, the research team decided to select only mid-size municipalities. These municipalities were initially contacted by email, outlining the purpose of the study and how it would be conducted. Then, if necessary, a phone call or a face-to-face meeting was organised to provide more details on the research project. Municipalities’ volunteered to participate in the project. Data on the characteristics of each municipality were collected from the regional health observatory database [45], including number of inhabitants, median income per inhabitant, number of people affected by a chronic illness, average number of new people each year affected by a chronic illness and number of written HEPA policies by sector. Then, department heads and elected officials from these municipalities who were involved in the sport, health and social sectors were invited to participate in the statement rating step. The aim of this participant selection was to have a range of perceptions about the factors related to HEPA policy development from several sectors. No participant was selected from the group of experts.

#### Statement rating

An individual meeting was scheduled with each participant in order to explain the aim of the study and statement rating instructions. Face-to-to face meetings increase the likelihood of participation from this type of informant. A scientific review showed that compliance of the rating step in CMA seemed to be higher with face-to-face meetings compared to those using the web-based Concept Systems Global Max^©^ software [43]. Participants were requested to complete paper-based surveys, including demographic information and expert-generated statements at their convenience. Collected participant demographic data included gender (man or woman), age (age category), physical activity level (a single question), training courses on physical activity and health (yes, no, or no but have knowledge), and number of written HEPA policies by sector. The participants were then asked to rate the importance and feasibility of each statement on a 6-point Likert scale. Studies on Likert scales have found that 4- to 7-point scales return the strongest reliability and validity [46, 47]. The advantage of 6-point scales is to avoid a midpoint forcing the choice [48]. Therefore, the choice was made to use a 6-point scale for statement rating. The participants were first instructed to rate the importance of each statement, independent of their local context, from (1) ‘not at all important’ to (6) ‘extremely important’. They were then instructed to rate the feasibility of each statement with regard to their local context, on the scale from (1) ‘not at all feasible’ to (6) ‘extremely feasible’. Once completed, paper-based surveys were sent to the first author. Data from these surveys were entered into the Concept Systems Global MAX^©^ software for analysis.

### Mapping analysis

Using Concept Systems Global MAX^©^ software, data from the statement rating step were computed to generate go-zone maps. Descriptive statistics on the importance and feasibility ratings were also calculated to create a go-zone map. Each point on the graph represented the mean rating value of each statement in terms of its importance and feasibility. The map was divided into four zones by the mean rating value of importance on the vertical axis and the mean rating value of feasibility on the horizontal axis. The upper right zone comprised the statements that were above the mean rating values of importance and feasibility; therefore, these statements were assumed to refer to potential levers. The upper left zone comprised those statements that were above the mean rating value of feasibility but below the mean rating value of importance. The bottom right zone contained the statements that were above the mean rating value of importance but below the mean rating value of feasibility. These statements could thus be assumed to refer to potential barriers. The bottom left zone comprised the statements that were below the mean rating values of importance and feasibility. The Concept Systems Global Max^©^ generates go-zone maps according to different scenarios. Go-zone maps were produced according to the sector (i.e. sport, health or social) and the position (i.e. department head or elected official) of the participants. The same and different HEPA policy levers and barriers between the sectors and the participants’ position were also identified.

## Results

A total of 84 statements sorted into 16 categories were generated. The rating participation by the municipalities was 65% (11/17). Table 1 presents the characteristics of the municipalities included in this study.

**Table 1:** Characteristics of the municipalities included

Municipality	Inhabitants (*n*)^a^	Median income (€)^b^	People affected by a chronic illness (*n*)^c^	Additional chronic illness (*n*)^d^	Number of written HEPA policies by sector (*n*)
A	74.875	22.392	12,441	2039	Sport (*n* = 2), Health (*n* = 1), Social (*n* = 1)
B	74.285	18.962	14,369	2237	Sport (*n* = 1), Health (*n* = 1), Environment (*n* = 1)
C	64.903	18.656	11,305	1837	Sport (*n* = 1), Health (*n* = 1)
D	50.937	20.704	7607	1230	None
E	49.322	22.046	8012	1305	Sport (*n* = 1)
F	41.571	20.010	7250	1102	None
G	35.296	23.152	6913	1088	None
H	28.919	22.858	4592	756	Sport (*n* = 2)
I	25.047	20.940	4656	780	Sport (*n* = 1), Health (*n* = 1)
J	23.347	21.778	3342	574	None
H	22.360	22.666	4047	674	None

*HEPA* Health-Enhancing Physical Activity

^a^Data from the National Institute of Statistics and Economic Studies - INSEE (2018) ; ^b^Data from INSEE (2018); ^c^Number of people affected by a chronic illness covered by governmental insurance for their healthcare expenditure. Data from the Regional Observatory of Provence-Alpes-Côte d’Azur (2018); ^d^Average number of additional people each year affected by a chronic illness covered by governmental insurance for their healthcare expenditure (from 2007 to 2014). Data from the Regional Observatory of Provence-Alpes-Côte d’Azur (2018)

The statements were rated by 33 key informants from 11 municipalities, including department heads (*n =* 23) and elected officials (*n =* 10). Key informants were from the sport (*n =* 14), health (*n =* 10) and social (*n =* 9) sectors. The demographic characteristics of the key informants are presented in Table 2.

**Table 2:** Demographic characteristics of the participants

	Overall *n* = 33 (%)	Sport *n* = 14 (%)	Health *n* = 10 (%)	Social *n* = 9 (%)	Department Heads *n* = 23 (%)	Elected Officials *n* = 10 (%)
**Sex**						
Men	14 (42)	10 (71)	1 (10)	3 (34)	10 (43)	5 (50)
Women	19 (58)	4 (29)	9 (90)	6 (66)	13 (57)	5 (50)
**Age categories**						
<30 years	0 (0)	0 (0)	0 (0)	0 (0)	0 (0)	0 (0)
30–39 years	6 (18)	2 (14)	1 (10)	2 (22)	6 (26)	0 (0)
40–49 years	3 (10)	1 (7)	2 (20)	2 (22)	2 (9)	1 (10)
50–59 years	18 (54)	10 (65)	5 (50)	3 (34)	14 (61)	4 (40)
60–69 years	3 (9)	0 (0)	1 (10)	1 (11)	1 (4)	2 (20)
≥70 years	3 (9)	1 (7)	1 (10)	1 (11)	0 (0)	3 (30)
**Have participated in a physical activity or health training course**						
Yes	9 (28)	6 (44)	2 (20)	1 (11)	7 (30)	2 (20)
No	12 (36)	4 (28)	2 (20)	6 (67)	9 (40)	3 (30)
No, but have knowledge	12 (36)	4 (28)	6 (60)	2 (22)	7 (30)	5 (50)
**Physical activity practice**						
No	4 (12)	0 (0)	1 (10)	3 (34)	2 (9)	2 (20)
Occasionally	11 (33)	4 (28)	4 (40)	4 (44)	7 (30)	4 (40)
Regularly	11 (33)	7 (50)	3 (30)	1 (11)	9 (40)	2 (20)
Often	4 (12)	2 (15)	2 (20)	0 (0)	4 (17)	0 (0)
Very often	3 (10)	1 (7)	0 (0)	1 (11)	1 (4)	2 (20)

### Overall group results

**Fig. 1:**
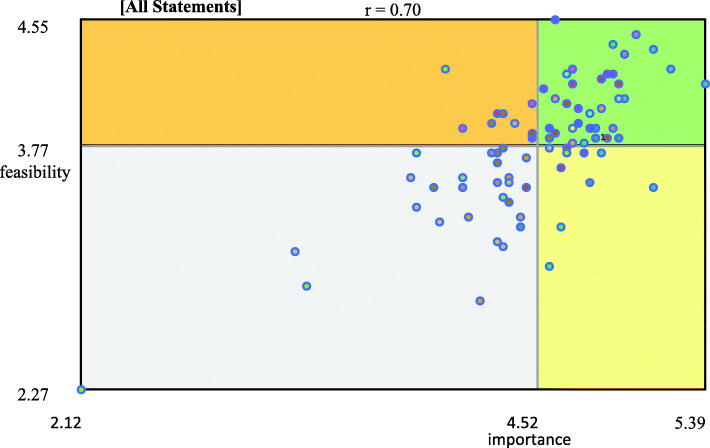
Go-zone map of the overall group

From the statement ratings by all participants (overall group results), the mean importance score was 4.52 (SD = 0.86) and the mean feasibility score was 3.77 (SD = 0.74). Figure 1 maps the potential levers (*n =* 38) (upper right zone) and barriers (*n =* 10) (bottom right zone) of HEPA policy development through go-zone analysis. Other statements were mapped in the upper left zone (*n =* 9) and bottom left zone (*n =* 27). Table 3 presents the top 10 statements identified as potential levers of and barriers to HEPA policy development. Table 4 presents the mean (M) and standard deviation (SD) of the rating of importance and feasibility by categories for overall group.

**Table 3:** Top 10 statements identified as potential levers of and barriers to HEPA policy development

Categories	Statements	M_***importance***_ (SD)	M_***feasibility***_ (SD)
**Levers**			
Population targeted	Target the community across the life course	5.39 (0.75)	4.15 (0.83)
Action on community	Develop communication strategies to inform, raise awareness and promote HEPA	5.03 (0.73)	4.45 (0.61)
Action on community	Develop events to inform, raise awareness and promote HEPA	4.97 (0.73)	4.33 (0.64)
Partnership	Partner with the sport sector	4.94 (0.83)	4.15 (0.56)
Human resources	Training for human resource personnel	4.94 (0.79)	4.06 (0.66)
Action on environment	Develop public spaces	4.94 (0.70)	3.82 (0.63)
Action on community	Identify the needs of the community	4.91 (0.91)	3.88 (0.89)
Practice targeted	Act on the school environment	4.91 (0.68)	4.39 (0.90)
Knowledge	Have knowledge about the diversity of local stakeholders that may be involved	4.91 (0.68)	4.21 (0.65)
Knowledge	Have knowledge based on field experience	4.88 (0.86)	4.21 (0.78)
**Barriers**			
Population targeted	Target vulnerable people (health)	5.12 (0.74)	3.52 (0.75)
Population targeted	Target disadvantaged people (social)	4.85 (0.87)	3.73 (0.67)
Action on environment	Develop active transportation	4.79 (0.80)	3.55 (0.66)
Knowledge	Have local contexts knowledge	4.76 (0.61)	3.73 (0.83)
Coordination	Have coordination mainly ensured by transversal relations between the departments	4.67 (1.19)	3.73 (0.80)
Mandate	Initiate actions beyond the duration of the mandate	4.67 (0.85)	3.76 (0.87)
Practice targeted	Act on active transportation	4.64 (0.82)	3.27 (0.63)
Mandate	Have policies consistent with those from other local governments	4.64 (0.89)	3.76 (0.65)
Practice targetedEconomic model	Act on the private sector Have an economic model involving other public funding	4.58 (0.75) 4.58 (0.66)	3.03 (0.64) 3.76 (0.56)

Note: overall group data (*n =* 33)

*HEPA* Health-Enhancing Physical Activity, *M* mean

**Table 4:** Mean (M) and standard deviation (SD) of the rating of importance and feasibility by categories for overall group

Categories	M_***importance***_ (SD)	M_***feasibility***_ (SD)
Mandate	4.63 (0.03)	3.93 (0.04)
Political commitment	4.31 (0.02)	3.72 (0.03)
Governance	4.75 (0.01)	3.99 (0.02)
Coordination	3.60 (0.73)	3.37 (0.48)
Population targeted	5.11 (0.03)	4.01 (0.09)
Practice targeted	4.71 (0.01)	3.70 (0.25)
Expression of the community	4.43 (0.05)	4.05 (0.02)
Knowledge	4.70 0.02)	4.09 (0.06)
Human resources	4.29 (0.01)	3.35 (0.11)
Expertise	4.46 (0.12)	3.70 (0.06)
Economic model	4.10 (0.14)	3.44 (0.06)
Action on community	4.60 (0.14)	3.91 (0.13)
Action on environment	4.76 (0.03)	3.67 (0.05)
Action on the organisation	4.70 (0.01)	3.89 (0.01)
Partnership	4.67 (0.03)	3.87 (0.04)
Evaluation	4.65 (0.03)	3.81 (0.04)

### Subgroup results

**Fig. 2:**
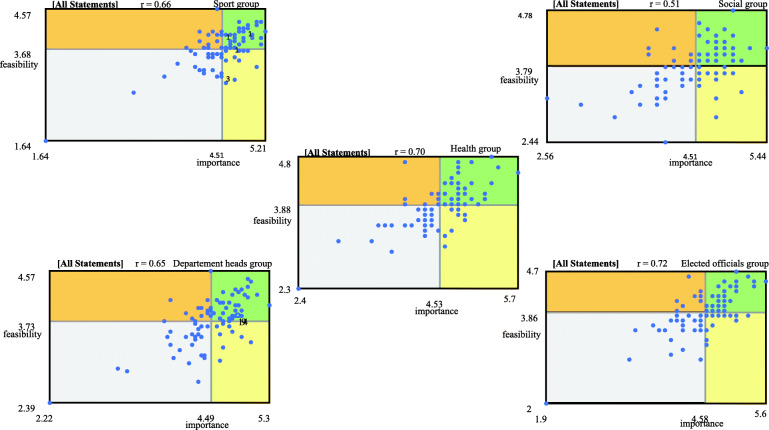
Go-zone map of the subgroups

In Table 5, the mean ratings of importance and feasibility are presented for the subgroups of sport, health, social, department heads and elected officials. Figure 2 presents the perceptions of levers and barriers to HEPA policy development according to subgroup. The numbers of potential levers and barriers were different according to the subgroup: sport (*n*_levers_ = 36, *n*_barriers_ = 17), health (*n*_levers_ = 40, *n*_barriers_ = 10), social (*n*_levers_ = 33, *n*_barriers_ = 18), department heads (*n*_levers_ = 34, *n*_barriers_ = 10) and elected officials (*n*_levers_ = 32, *n*_barriers_*=* 18).

**Table 5:** Mean (M) and standard deviation (SD) of the rating of importance and feasibility for subgroups

Subgroups	M_***importance***_ (SD)	M_***feasibility***_ (SD)
Sport sector	4.53 (0.76)	3.88 (0.68)
Health sector	4.45 (0.83)	3.68 (0.74)
Social sector	4.51 (0.83)	3.79 (0.72)
Department Head	4.49 (0.84)	3.73 (0.71)
Elected Official	4.58 (0.88)	3.86 (0.78)

Additional file 1 shows the sector and position of each respondent for each statement identified as a potential lever or barrier. The same statements were identified as potential levers (*n* = 17) across the sport, health and social groups, whereas no statement was unanimously rated as a potential barrier by these groups. When we considered the positions of respondents, the same statements were identified as potential levers (*n* = 21) and potential barriers (*n* = 4) by both department heads and elected officials.

Additional file 2 presents the descriptive statistics for all statements and categories according to subgroup.

## Discussion

This study captured perceived barriers and levers of HEPA policy development from elected officials and department heads in the sport, health and social sectors of French municipalities.

All municipalities included in this study are considered mid-size in France (between 20,000 and 100,000 residents [44]). While the size of municipalities was standardised to the selection, there is still an important size disparity between some of them, which could possibly influence the perception of policy-makers on HEPA policy development. However, municipalities that have almost the same number of residents could also be quite different due to environmental characteristics (e.g. presence and accessibility of public spaces, parcs, walking and cycling paths, etc.) or the population (e.g. lifestyle, social inequalities, etc.). Thus, these characteristics could influence the perception of policy-makers to develop policies. As the municipality is a complex ecosystem [49, 50], it is difficult to select a homogeneous sample. Further research should identify the main municipality characteristics that could influence HEPA policy development.

Some studies have also shown that the individual characteristics of policy-makers can influence their perceptions regarding policies promoting HEPA, including their personal physical activity practice [51, 52]. In this study, most participants declared to occasionally or regularly practice some form of physical activity or to have knowledge or training in physical activity and health. However, a big gap remains between perception, priority and action. Policy-makers may engage in physical activity or perceive its positive effects on health but not prioritise it, which limits the development of HEPA policy.

These findings helped to identify potential levers and barriers to policy development. Some of the potential levers, such as ‘target the community across the life course’ (statement 23) and ‘develop public spaces’ (statement 66), were in line with the HEPA recommendations of WHO [53, 54]. Other statements, such as ‘develop active transportation’ (statement 67) or promote HEPA for ‘vulnerable people’ (statement 27) and ‘socially disadvantaged people’ (statement 28), were perceived as important and in line with the WHO recommendations but were not considered feasible (potential barriers).

The barriers identified in this study seemed to be due to local factors. The findings showed a gap between what was rated as important for developing HEPA policies when the local context was not considered and what was rated as feasible in the local context. According to the literature, HEPA promotion should be adapted and embedded in context [2, 30]. Nevertheless, these results suggest that it is sometimes difficult to adapt HEPA policy-making to real contextual settings in a complex ecosystem like a municipality.

The finding of barriers suggests that municipalities need to be supported in their efforts to overcome them. More research in other contexts is needed to observe if these barriers are recurring and to better understand why they are not overcome. However, one difficulty is that barriers seem to differ according to the policy-maker’s sector and position, as shown by the results. Thus, we hypothesise that a framework standardising the collection of qualitative data on local HEPA policy development and analyses that takes into account the policy-makers’ characteristics would provide evidence on how best to support municipalities.

The levers for HEPA policy development also varied according to the sector and position of the policy-makers. However, concept mapping might be an interesting way for them to encourage and enhance intersectoral collaborations inside and outside a given municipality, as recommended in the literature [13, 14, 16]. This approach can highlight a shared vision and the potential levers that are common to the various sectors. Yet, in order to make headway in adopting intersectoral policies, the policy-makers from these sectors need to share not only a vision and levers, but also a common language and policy goals [18]. Moreover, it may not be enough to involve key stakeholders from other sectors in HEPA promotion. Studies have highlighted that the awareness of HEPA importance by elected officials and department heads from various sectors determine their involvement in HEPA promotion [25, 26]. Strong leadership and strong political advocacy might therefore help enhance the development of intersectoral HEPA polices [53, 54]. The use of concept mapping by municipalities might also highlight the differing perceptions across sectors on the importance and feasibility of developing local HEPA policies. This would shed light on why sectors sometimes choose to work in ‘silos’ rather than collaborate.

This study had some limitations. The research was restricted to France and the mid-size municipalities were from only two counties both in the southern region. Thus, the generalisability of these results is limited. Furthermore, it is highly likely that the municipalities that volunteered to participate in this study were more involved in HEPA promotion. Due to difficulties in recruiting policy-makers from municipalities to participate in several steps of CMA, the methodology was adapted. This could be considered a limitation. The brainstorming and rating steps were made by two different groups. The sample size of the expert group who generated and sorted statements was small. Some relevant statements might therefore have been missed in the list proposed by the expert group. Moreover, as statements were not sorted into categories using Concept Systems Global MAX^©^ software, some statistical analysis could not be done. Policy-makers who participated in this study were only from three sectors, although many other sectors, such as the urban, environmental or educational sectors, could be involved in HEPA promotion. Therefore, it was not possible to analyse every position in every sector. Similarly, it was not possible to analyse the perceived barriers and levers of HEPA policy according to the characteristics of municipalities. The statement ratings were based on perceptions and this as well might have biased the results due to social and political desirability [55]. In addition, the ratings might have been influenced by the participants’ personal physical activity levels, their knowledge about physical activity and health, or by the characteristics of their municipality. Last, some of the statements that emerged or did not emerge from the go-zone as potential levers or barriers might be explained by threshold effects.

## Conclusions

This study contributes to a better understanding of the development of local HEPA policies. It does so by capturing and analysing the perceptions of key informants about local HEPA policy development in mid-size French municipalities. The findings revealed potential levers and barriers. According to the sector (sport, health, social) and the position (department heads, elected officials), some of these potential levers and barriers were shared and others were informant specific. Although sector-related perceptions can affect intersectoral collaboration, the use of concept mapping by the local government might counter this tendency and enhance collaboration. Findings also showed a gap between what the policy-makers deemed important to do to develop HEPA policies when local context was not considered and what they thought was feasible in their local context. The results indeed suggested that local context factors might affect the development of HEPA policies in mid-size French municipalities. Findings further suggested that municipalities need to be supported to overcome barriers and more easily develop HEPA policies in local contexts. To this end, collecting local HEPA policies from a large sample of municipalities using a standardised framework could help to compare and better understand these policies. Thus, analysing qualitative data such as the type and the content of a HEPA policy as well as the characteristics of the local context, would likely provide evidence to support municipalities in their policy-making. More research is now needed to extend the analysis of local HEPA policies in different local and country contexts.

## Supplementary information

**Additional file 1.**

**Additional file 2.**

## Data Availability

All data generated or analysed during this study are included in this published article and its supplementary information files.

## Abbreviations

HEPA: Health-Enhancing Physical Activity
CMA: Concept Mapping Approach
