# Supplementary material for: Perceptions of barriers and levers of health-enhancing physical activity policies in mid-size French municipalities
Source: Health Res Policy Syst. 2020 Jun 8;18:62. doi: 10.1186/s12961-020-00575-z (PMC7278119; doi:10.1186/s12961-020-00575-z)
Supplement: Supplementary file 1 — Additional file 1. [file 12961_2020_575_MOESM1_ESM.docx]

**Supplement 1. List of statements identified as potential levers and barriers for HEPA policy development.**

| **Statements** | **Levers** | **Barriers** |
| --- | --- | --- |
|  |  |  |
| 1. State disease prevention and health promotion of inhabitants as a priority. | H, S, DH, EO |  |
| 2. Consider the improvement in the PA level of inhabitants as a major lever of disease prevention and health promotion. | SP, H, S, DH |  |
| 3. Try to overcome the constraints of elected officials. |  |  |
| 4. Initiate actions beyond the duration of the mandate. | SP, H, DH | S, EO |
| 5. Have policies consistent with those of other local governments. | EO | H, S, DH |
| 6. Have a clear political commitment that is displayed in the campaign program. |  |  |
| 7. Have a political commitment resulting in strong and permanent leadership. |  | H |
| 8. Have a political commitment formalized by documents, frameworks and/or development plans. |  |  |
| 9. Have a political commitment resulting in the reorganization of services. |  |  |
| 10. Have a political commitment resulting in the reorganization of collaborations between departments. | H | S, DH |
| 11. Have a political commitment resulting in new and specific missions for elected officials on this topic. |  |  |
| 12. Have a political commitment resulting in the reallocation of financial resources. |  | EO |
| 13. Have a political commitment resulting in the reallocation of human resources. | SP |  |
| 14. Have a governance that allows for the elaboration of a shared project between the elected officials from various sectors. | SP | H, S, DH, EO |
| 15. Have a governance that allows for the elaboration of a shared project between the department heads from various sectors. | SP, S, EO | H, DH |
| 16. Have a governance that allows for the elaboration of a project shared between the elected officials and department heads. | SP, H, S, EO | DH |
| 17. Consult with the staff of all involved departments during the planning and implementation of a project. | SP, S, EO | DH |
| 18. Have the coordination mainly led by the sports department. |  |  |
| 19. Have the coordination mainly led by the health department. |  |  |
| 20. Have the coordination mainly led by another department. |  |  |
| 21. Have the coordination mainly led by a dedicated structure. |  |  |
| 22. Have the coordination mainly ensured by transversal relations between the departments. | H | S, DH, EO |
| 23. Target the community across the life course. | SP, H, S, DH, EO |  |
| 24. Target children and teenagers. | SP, H, S, DH, EO |  |
| 25. Target adults. | SP, H, S, DH, EO |  |
| 26. Target elderly people. | SP, H, S, DH, EO |  |
| 27. Target vulnerable people (health). | H, | SP, S, EO |
| 28. Target disadvantaged people (social). | H, S, EO | SP, DH |
| 29. Act on active transportation. |  | SP, H, DH, EO |
| 30. Act on the school environment. | SP, H, S, DH, EO |  |
| 31. Act on leisure activities outside sports clubs. | S, EO | SP, H, DH, |
| 32. Act on leisure activities in sports clubs. | SP, H, S, DH, EO |  |
| 33. Act on the private sector. |  | H, S, DH |
| 34. Promote the participative approach of the community by enabling direct communication with the municipality. | H, S, EO |  |
| 35. Promote the participative approach of the community through citizen committees. |  |  |
| 36. Promote the participative approach of the community through associations, neighbourhood committees or other organized networks. | SP, H, S, EO |  |
| 37. Have local contexts knowledge. | H | SP, S, DH, EO |
| 38. Have knowledge about the diversity of local stakeholders that may be involved. | SP, H, S, DH, EO |  |
| 39. Have knowledge about different departments in the municipality. | H, S, EO |  |
| 40. Have knowledge about other public services. | SP, H, DH |  |
| 41. Have knowledge based on field experience. | SP, H, S, DH, EO |  |
| 42. Have scientific knowledge on this topic. | SP |  |
| 43. Have knowledge of laws, plans and recommendations on this topic. | SP, H, S, DH, EO |  |
| 44. Recruit additional human resources. |  | SP |
| 45. Reassign human resources. |  |  |
| 46. Mutualize human resources between departments in the municipality. | H, EO | S |
| 47. Mobilize volunteer human resources. |  | S |
| 48. Training for human resource personnel. | SP, H, S, DH | EO |
| 49. Rely on a HEPA expertise centre. |  |  |
| 50. Rely on expert-volunteers. |  | S |
| 51. Allocate specific financial resources. |  | SP, H |
| 52. Have an economic model from the private sector. |  |  |
| 53. Have an economic model based on the municipality's own resources. |  |  |
| 54. Have an economic model involving other public funding. | EO | DH |
| 55. Have an economic model based on public-private solutions. |  |  |
| 56. Have an economic model based on public health insurance. |  | SP, EO |
| 57. Have an economic model based on private health insurance. | EO | SP |
| 58. Have an economic model based on the financial participation of users. |  |  |
| 59. Have an economic model with a social tariff. | S | EO |
| 60. Identify the needs of the community. | SP, H, DH | S, EO |
| 61. Set up a local HEPA program ensured by the municipality. | SP |  |
| 62. Set up a place to centralize information about local opportunities. |  | SP |
| 63. Develop an assessment and orientation service towards local HEPA programs. |  | SP |
| 64. Develop communication strategies to inform, raise awareness and promote HEPA. | SP, H, S, DH, EO |  |
| 65. Create events to inform, raise awareness and promote HEPA. | SP, H, S, DH, EO |  |
| 66. Develop public spaces. | H, S, DH | SP, EO |
| 67. Develop active transportation. | S | SP, H, DH |
| 68. Create sports facilities. |  | SP |
| 69. Maintain or renovate sports facilities. | SP, H, DH, EO | S |
| 70. Provide time slots in existing sports facilities. | H, S, DH | SP, EO |
| 71. Support the stakeholders who propose local HEPA programs. | SP, S, DH | EO |
| 72. Coordinate a local stakeholder’s network. | SP, H, DH, EO | S |
| 73. Subsidize associations that offer local HEPA programs. | SP, S, DH |  |
| 74. Partner with the health sector. | H, S, DH | SP, EO |
| 75. Partner with the social sector. | H, S, EO | SP |
| 76. Partner with the sports sector. | SP, H, S, DH, EO |  |
| 77. Partner with the local associations. | H, S | EO |
| 78. Partner with the school sector. | SP, H, S, DH | EO |
| 79. Partner with the private sector. | H | EO |
| 80. Evaluate actions according to the campaign program. | SP, H, EO |  |
| 81. Evaluate actions based on efficiency indicators. | SP, DH, EO | S |
| 82. Evaluate the return on investment of interventions. |  | S |
| 83. Evaluate the follow-up of interventions. | SP, H, DH, EO | S |
| 84. Evaluate the sustainability of interventions. | SP, H, DH, EO | S |
|  |  |  |

Note: subgroups: Sport sector (SP); Health sector (H); Social sector (S); Department Head (DH); Elected Official (EO).
